# Mouse Vestibulo-Ocular Reflex Testing for Otolith Organs and Horizontal Semicircular Canal

**DOI:** 10.21769/BioProtoc.5509

**Published:** 2025-11-20

**Authors:** Tong Zhao, Shijie Xiao, Qingsong Liu, Jinxuan Liu, Fangyi Chen

**Affiliations:** 1 Department of Biomedical Engineering, Southern University of Science and Technology, Shenzhen, China; 2 Giant Technologies Co., Ltd, Shenzhen, China

**Keywords:** Vestibulo-ocular reflexes (VORs), Comprehensive mouse vestibular function evaluation, Semicircular canals, Otolith organ, Off-vertical axis rotation (OVAR)

## Abstract

Vestibulo-ocular reflexes (VORs) are compensatory ocular reflexes that maintain stable vision during head movements. In research, VORs encompass angular VOR (aVOR) and off-vertical axis rotation (OVAR) tests, which various groups have employed to assess vestibular function in mice. This protocol outlines the process for measuring VORs in mice, including eye rotation calibration, immobilizing the mouse with a noninvasive setup, configuring the aVOR and OVAR stimulus modes, and interpreting the obtained waveforms to derive VOR values. As technology advances, VORs are expected to yield more qualitative and quantitative insights into the function of the horizontal semicircular canal cristae (HSCC) and the otolith organs. This methodology can serve as a standard for evaluating common vestibular deficits in mice.

Key features

• The integrated aVOR and OVAR modes enable us to evaluate both the otolith organs and horizontal semicircular canals.

• The calibration tools used in our system ensure standardization between different systems, facilitating comparison of results between laboratories.

• Animal holders provide a rapid and convenient method for conducting VOR tests without the need for anesthesia or surgery.

## Graphical overview


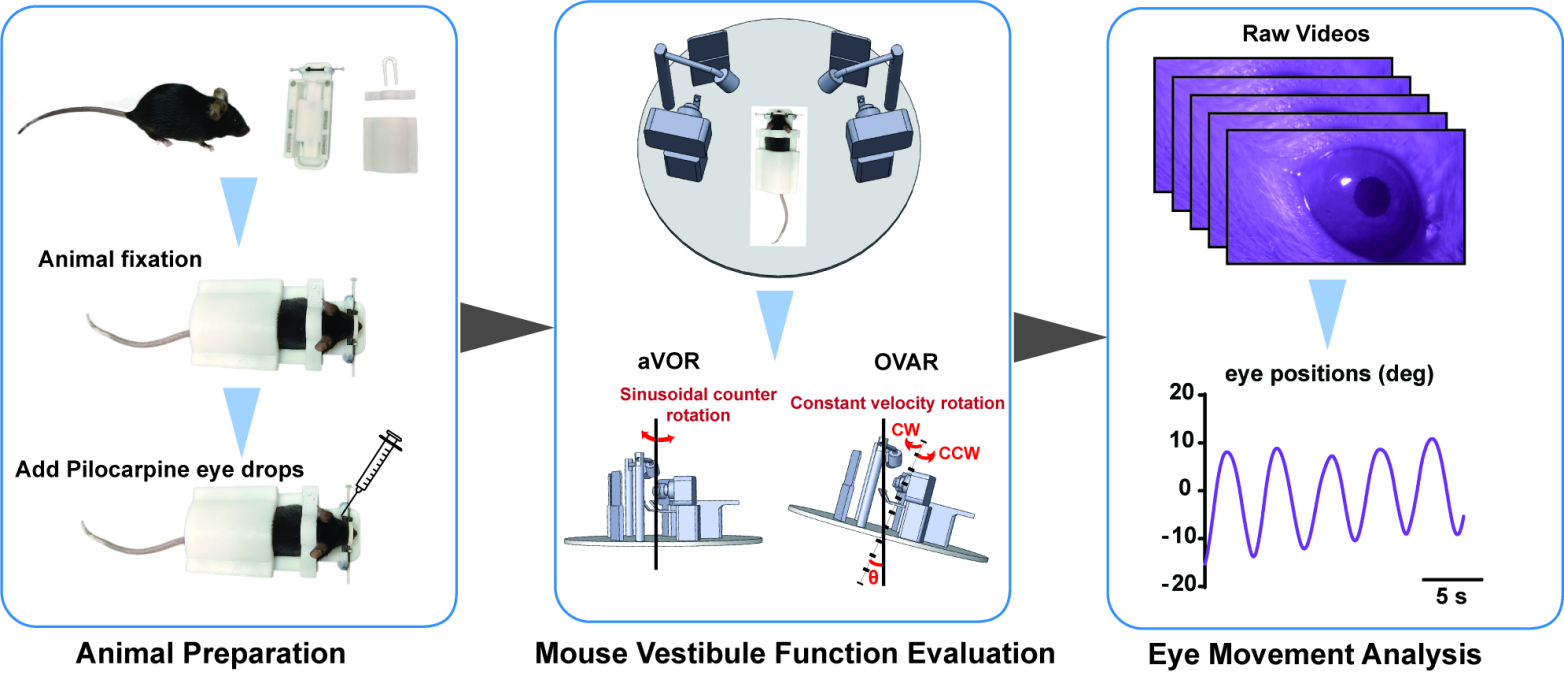


## Background

The vestibular system detects changes in head and body position, controls gaze, and facilitates higher-level cognitive perception functions. It comprises three semicircular canals and two otolith organs (the utricle and saccule), which mediate sensitivity to rotational and linear (including gravitational) head movements, respectively. Vestibulo-ocular reflexes (VORs) are commonly used in clinical testing [1] and have also been part of standard vestibular function tests in primate studies [2–4]. VORs can be classified into angular VOR (aVOR), induced by rotational accelerations and primarily driven by signals from horizontal semicircular canal cristae (HSCC) [5,6], and translational VORs (tVORs), such as linear VOR (LVOR) and off-vertical axis rotation (OVAR), which provide otolith stimulus [7,8]. OVAR typically entails constant rotational velocity of the mouse head about an axis that is tilted to the Earth's vertical axis. After achieving steady state rotation, the semicircular canal response decays, and the resulting ocular response originates from otoliths [2,7]. LVOR response is too small to provide reliable estimates of otolith function [9], but OVAR signals are sufficiently robust, and OVAR stimulus could be easily combined with aVOR stimulus into one machine. Therefore, the combination of aVOR and OVAR can form the basis of a complete functional test for the peripheral vestibular organ. The purpose of this protocol is to provide a detailed procedure to encourage further study of the vestibular system's function.

## Materials and reagents

**Reagents**

1. 0.5% Pilocarpine eye drops (Bausch + Lomb, ZhenRui@)

2. Stroke-physiological saline solution (Beyotime, catalog number: ST341-500 mL)

**Laboratory supplies**

1. 1 mL syringe (Beyotime, catalog number: FS801-180pcs)

## Equipment

1. Noninvasive animal-immobility setup (Shenzhen Giant Tek Co., Ltd, catalog number: GT-MHOLDER1035)

2. VOG-based VOR test system (Shenzhen Giant Tek Co., Ltd, catalog number: GT-MVOR03)

3. Eye rotation calibration tools (Shenzhen Giant Tek Co., Ltd, catalog numbers: GT-MVCA01, GT-MVCA02, GT-MVCA03)

## Software and datasets

1. Eye movement data processing software (Shenzhen Giant Tek Co., Ltd, catalog number: GT-MVORSW1.0). The software was created using MATLAB (2021b)

## Procedure

**A. Instrument preparation**

1. Turn on the instrument and wait 2–3 s for the camera to power up synchronously.

2. Connect a tablet to the camera's Wi-Fi to display the real-time image. (**Critical**: The pupils of mice can be viewed even in the dark.)

**B. Animal preparation**

**Figure 1.**
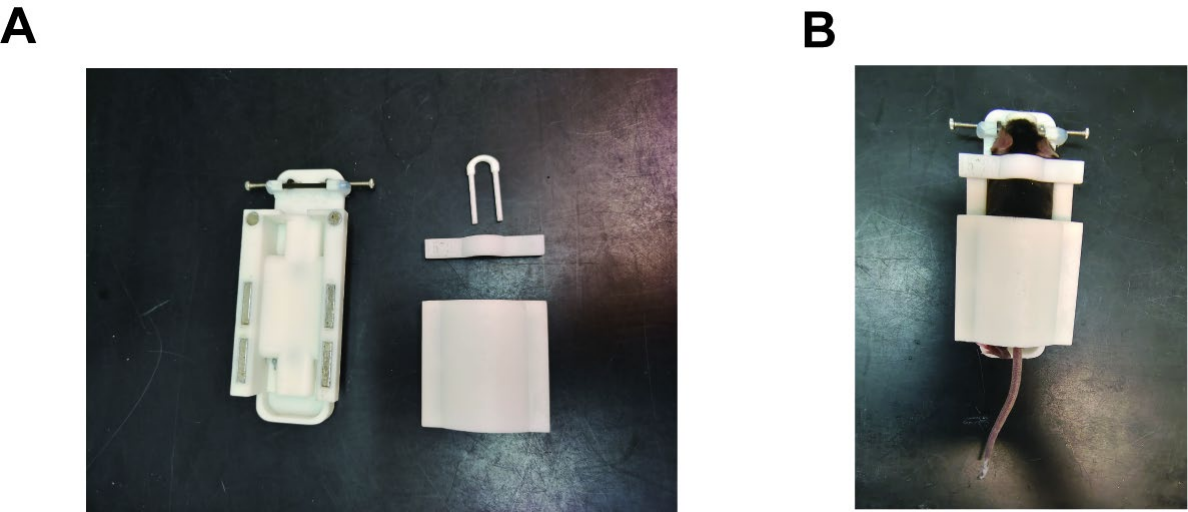
Noninvasive animal-immobility setup. (A) The noninvasive animal-immobility setup consists of four magnetic parts: a holder base, a nose holder, a neck holder, and a back holder. (B) In the setup, mice are head-fixed at ~30° nose-down to maintain the horizontal semicircular canals parallel to the yaw plane.

1. Animal fixation: The noninvasive animal-immobility setup consists of four magnetic parts: a holder base, a nose holder, a neck holder, and a back holder (Figure 1A).

a. Position the awake mouse on the holder base.

b. Sequentially attach the neck holder, back holder, and nose holder to the holder base to fix the mouse (Figure 1B).

**Caution**: When working with mice, gloves and a lab coat should be worn at all times. Additionally, for less experienced users, a heavy glove may be worn when grabbing older mice to protect from biting.

*Notes:*

*1. We do not recommend the use of anesthesia, even for a short period, as it affects eye movement.*

*2. The neck holder should not be too tight, or it can interfere with eye movement.*

2. Add Pilocarpine eye drops.

a. After fixation, apply 1–2 μL of pilocarpine eye drops to each of the mouse’s eyes using a 1 mL syringe to induce pupil constriction for testing in the dark.

b. Once miosis is achieved, gently wipe away any excess eye drops with a soft tissue soaked in saline.

*Note: The mouse will be ready for testing after approximately 5 min for the eye drops to take effect. Appropriate levels of miosis result in the pupil size in a dark environment being the same as that in a normal light environment.*

**C. Mouse vestibule function evaluation**

The integrated and surgery-free instrument system contains angular VOR (aVOR) and off-vertical axis rotation (OVAR) modes for evaluating mouse vestibular function. The instrument parameters of aVOR and OVAR are shown in Table 1.

1. Adjust camera focus:

a. Position the fixed and miotic mouse on the instrument.

b. Fine-tune the distance of the camera in the front, back, left, and right directions to ensure the image of the mouse’s eyes is clear and centered.

c. Close the lid of the instrument and keep the mouse in the dark.

**Figure 2.**
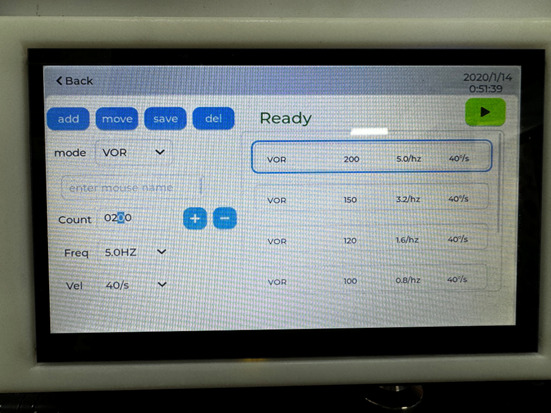
Electronic touch screen of the instrument. The screen allows users to set parameters (frequencies, time, and velocities) for both aVOR and OVAR. The *add* button enables the addition of a mode; the *move* button allows users to switch the order of modes; the *save* button stores the mode; and the *del* button deletes the mode.

2. Set instrument parameters:

a. aVOR. The aVOR is generally used to assess HSCC function [6]. During the HSCC test, the animal is stimulated by the sinusoidal oscillation of the platform at frequencies of 0.2, 0.5, 0.8, 1.6, 3.2, and 5.0 Hz, with peak velocities of 40°/s. Each stimulation lasts for at least 50 cycles.

i. Configure the parameters (frequencies, time, and velocities) of aVOR on the electronic touch screen of the instrument (Figure 2).

ii. Click to start stimulation, and the video camera will simultaneously record the mouse's eye movement. The eye movement during each test profile is recorded in a separate video file.

b. OVAR. The OVAR test has been used to evaluate otolith organ function in mice [5,10]. To accommodate the OVAR test, the equipment system is hinged to a 50 × 50 × 2 cm aluminum base, which holds a motorized push rod to raise the back of the system and create a pitch of set angles. During the OVAR test, the equipment is tilted at 17° or 30°. The platform rotates at constant velocities of 30, 50, and 80°/s in clockwise (CW) or counterclockwise (CCW) directions. Each stimulation lasts for 2–3 min.

i. Configure the parameters (frequencies, time, and velocities) of OVAR on the electronic touch screen of the instrument (Figure 2).

ii. Click to start stimulation; the video camera will simultaneously record the mouse's eye movement. The eye movement during each test profile is recorded in a separate video file.

3. After VOR measurements, open the nose holder, the neck holder, and the back holder in sequence and return the mouse to their original cage.

*Notes:*

*1. In general, a mouse will remain miotic for ~30–40 min after the first application of eye drops.*

*2. If the mouse's pupil starts dilating and the pupils are obscured by the eyelids, the VOR recording will be disrupted, and 1–2 μL of eye drops must be re-applied to the mouse.*

*3. Sections A–C are shown on the YouTube video:* *https://www.youtube.com/watch?v=YSHMsHDkunQ&list=LL* .

**D. Eye movement analysis**

**Figure 3.**
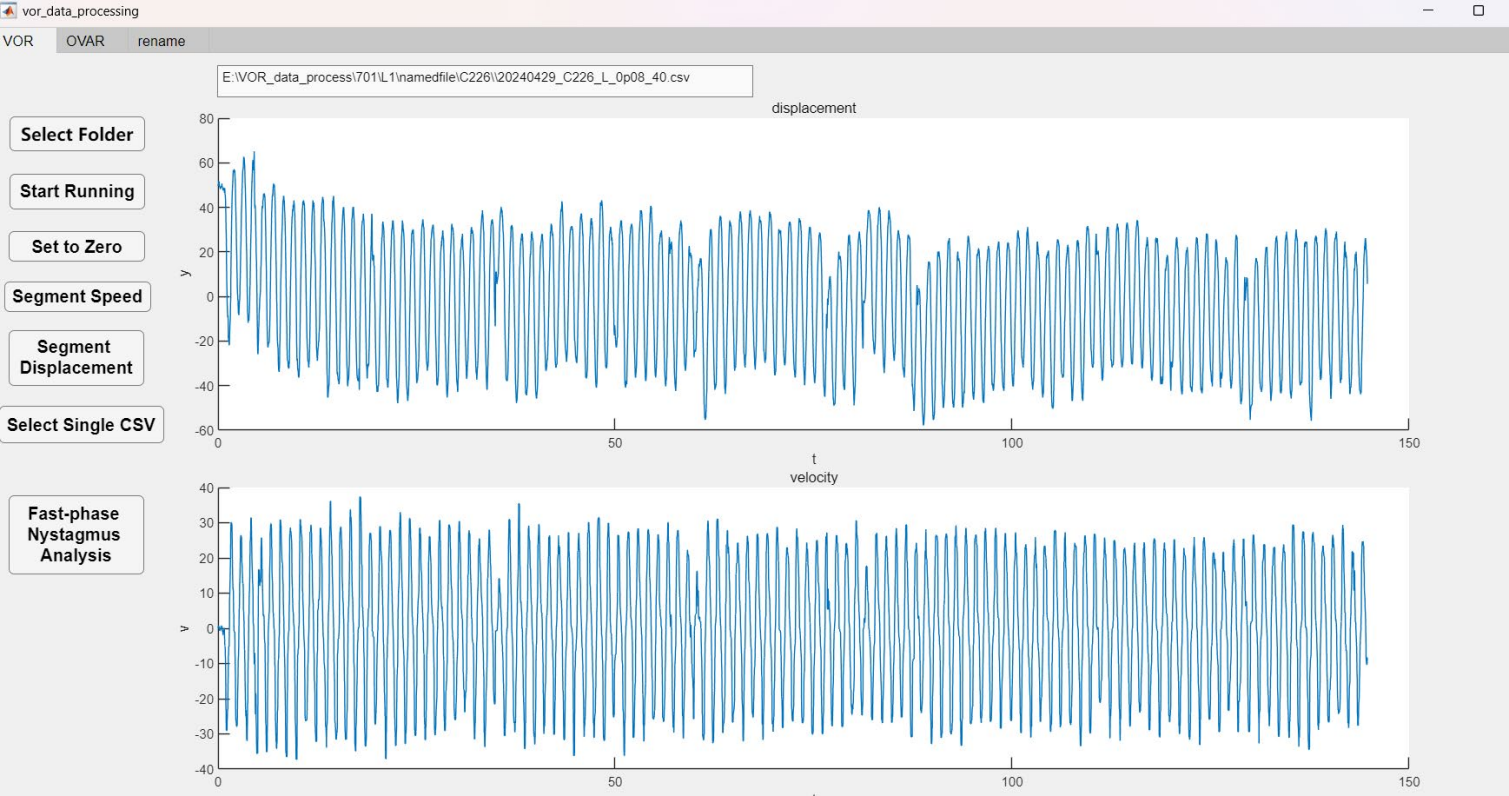
Eye movement data processing software. Typical aVOR waveforms of the control group (blue) were induced by sinusoidal rotation of 0.8 Hz, with a peak velocity of 40°/s.

1. Eye movement video processing: The recorded videos can be automatically downloaded via Wi-Fi to a PC or edge computing device for processing. At the end of the processing, an Excel file corresponding to each video is generated, containing the trajectory information of the eye movements.

2. Eye movement data processing:

a. Use the eye movement data processing software (Figure 3) to open the Excel file folder.

b. Select aVOR or OVAR option.

c. The software automatically reads and calculates the gain and phase of aVOR or OVAR threshold.

**Table 1.** Rotation modes of aVOR and OVAR.

	Frequency (Hz)	Angular velocity (°/s)	Direction
aVOR	0.1–10.0	10–80	Sinusoidal counter rotation
OVAR		10–80	Clockwise, counter-clockwise

## Validation of protocol

**Figure 4.**
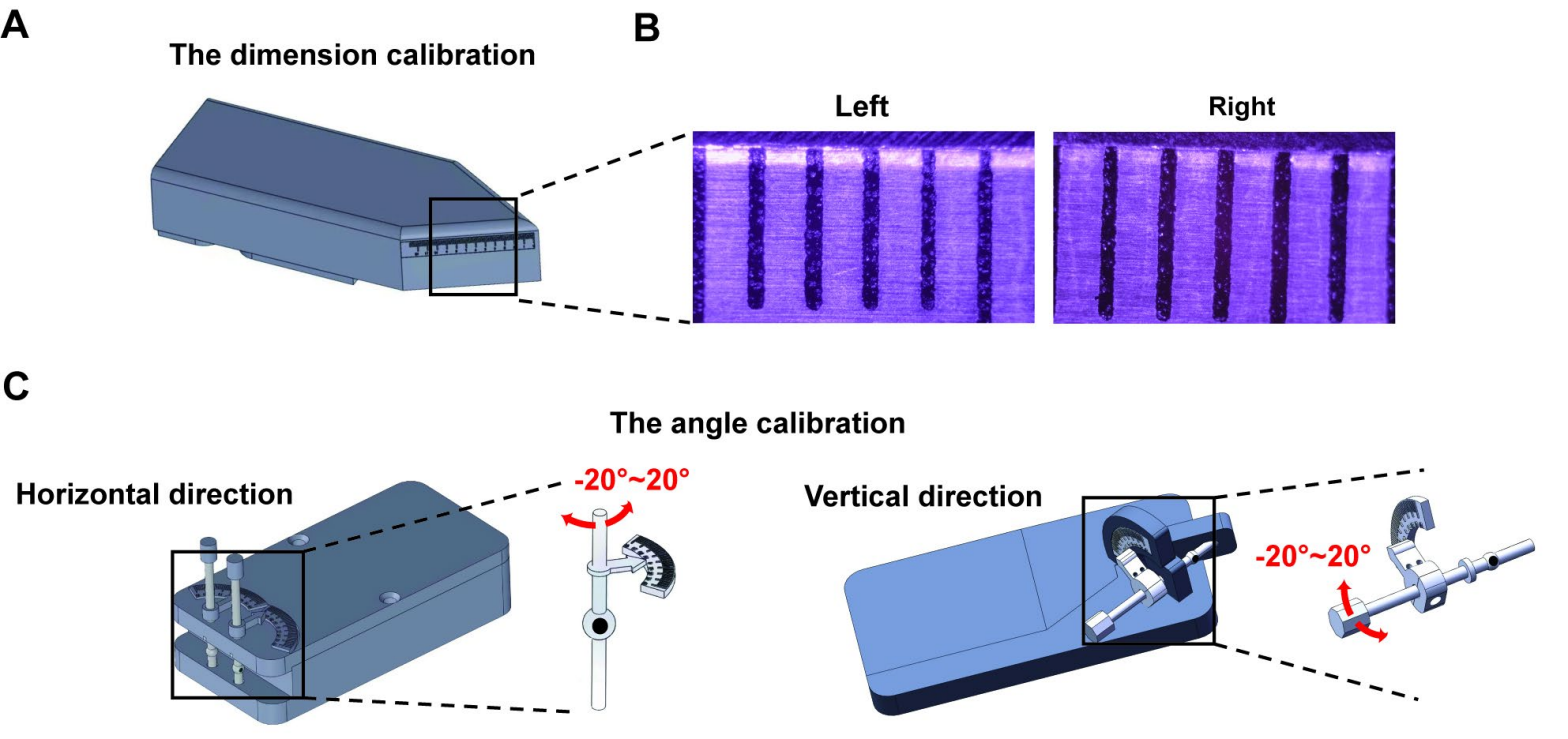
Eye rotation calibration. (A) Dimension calibration tool. (B) The zoom length of the two cameras in the system is adjusted to 6 mm. (C) Horizontal and vertical angle calibration of the eye movement recording system.

This protocol (or parts of it) has been used and validated in the following research article:

Xiao et al. [11]. Semicircular Canals Input Can Modify the Fast-Phase Nystagmus in Off-Vertical Axis Rotation of Mice. *eNeuro* (Figure 4a–c)

## General notes and troubleshooting

**General notes**

**A. Eye rotation calibration**

Eye rotation calibration tools are used to calibrate the zoom factor of the camera system and verify the accuracy of the eye movement analysis, ensuring repeatability across different systems and facilitating the comparison of results from various laboratories.

1. Dimension calibration

a. Place the dimension calibration tool (Figure 4A) on the instrument.

b. Adjust the zoom length of both cameras in the system to ensure that their fields of view are set to 6 mm (Figure 4B).

2. Angle calibration: The rotational angle of the eye movement is calibrated using an artificial eyeball model attached to a protractor.

a. Position the angle calibration tool (Figure 4C) on the instrument.

b. During calibration, rotate the artificial eyeball to precise angles using the protractor, while recording the image of the artificial eyeball at these various angles. The angular calibration scale can be calculated based on the translational distance of the pupil in the picture and the angular readout from the protractor.
